# Inequalities in esophageal cancer mortality in Brazil: Temporal trends and projections

**DOI:** 10.1371/journal.pone.0193135

**Published:** 2018-03-19

**Authors:** Juliano dos Santos, Karina Cardoso Meira, Taynãna César Simões, Raphael Mendonça Guimarães, Mauricio Wiering Pinto Telles, Laiane Felix Borges, Auzenda Conceição Parreira de Assis, Maria das Vitorias Silva, Isabelle Ribeiro Barbosa, Angela Carolina Brandão de Souza Giusti, Camila Alves dos Santos, Dyego Leandro Bezerra de Souza

**Affiliations:** 1 National Cancer Institute, Cancer Hospital III, Rio de Janeiro, Brazil; 2 Federal University of Rio Grande do Norte, Health School, Natal, Brazil; 3 Research Center René Rachou, Oswaldo Cruz Foundation, Rio de Janeiro, Brazil; 4 Joaquim Venâncio Polytechnic School, Oswaldo Cruz Foundation, Rio de Janeiro, Brazil; 5 Federal University of Rio Grande do Norte, Department of Collective Health, Graduate Program in Collective Health, Natal, Brazil; 6 Federal University of Rio Grande do Norte, Graduate program in Collective health, Natal, Brazil; Scientific Institute of Public Health (WIV-ISP), BELGIUM

## Abstract

The main objective of the study was to analyze the effect of age, period and birth cohort on esophageal cancer mortality in Brazil and its geographic regions, per sex. An ecological study is presented herein, which evaluated the deaths by esophageal cancer and the distribution, per geographic region. Poisson Regression was utilized to calculate the effects of age, period and birth cohort, and projections were made with the statistical software R, using the age-period-cohort model. Projection of data covered the period 2015–2029. Regarding the geographic regions of Brazil, a decrease was verified, throughout time, for the mortality rates of the South and Southeast regions, for men and women. For the North, Northeast and Midwest regions, an increase was evidenced in mortality rates, mainly for men, after the 2000's. Regarding the projections, a progressive increase of mortality rates was verified for the Northeast and North regions. Divergences evidenced for observed and projected esophageal cancer mortality rates revealed inequalities among the geographic regions of Brazil.

## Introduction

Esophageal cancer is the eighth most incident type of cancer in the world, and is the sixth cancer-related cause of death, presenting high lethality with global 5-year survival under 15%[1–3]. Disparities are highlighted in the distribution of this neoplasm around the world, as approximately 80% of cases and mortality occur in developing countries. In this context, the highest incidence and mortality rates are verified in Oriental Asia, Micronesia/Polynesia, and Oriental Africa, while intermediate rates are observed in South America and the Caribbean, and the lowest rates are found in North America, North Europe and Occidental Africa [1–2].

Squamous cell carcinoma (SCC) and adenocarcinoma (AC) are the two most incident histological types of esophageal cancer, which present different distributions throughout the world, in by sex, being most incident in men [4–8]. SCC is most incident in poor countries, and 83.1% of cases occur in Centro-Oriental Asia (79.0%) and Latin America (4.1%). In contrast, 46% of AC cases occur in developed countries located in Occidental Europe, North America and Oceania [8].

This disparity in the distribution of SCC and AC cases can be explained by the fact that these pathologies present differentiated risk factors in the carcinogenesis process. In esophageal carcinoma, risk factors that have been widely studied, with strong evidence of association include tobacco consumption, excessive consumption of alcoholic beverages, low ingestion of fruit and vegetable, exposure to occupational agents such as benzene, silica, asbestos, gamma radiation, rubber or paint industry, and formaldehyde [9–10]. Azevedo and Silva *et al*. (2016) verified a population attributable risk (PAR) for esophageal cancer and the aforementioned factors over 72.8% for men and 60.2% for women [11], and in both sexes, the highest contribution was from tobacco and alcohol consumption.

For esophageal adenocarcinoma carcinogenesis, the risk factors with strong evidence of association are gastroesophageal reflux, obesity, consumption of tobacco and Barrett's esophagus [9–10]. Olsen *et al*. (2011) found a 76% population attributable risk for the combination of tobacco consumption, gastroesophageal reflux, and obesity. The factors that contributed the most to PAR were gastroesophageal reflux and obesity [12].

Esophageal cancer incidence and mortality are strongly associated with low socioeconomic conditions of specific regions of the world [1–3,5]. In this sense, it is necessary to analyze the mortality rate evolution due to this disease considering territorial socioeconomic disparities, especially in countries scarred by inequalities in healthcare.

Brazil presents considerable social and healthcare inequalities across its geographic regions. This occurs because each geographic region has its own characteristics. The South region, for example, presents the best indices related to human development. The richest and most populated region is the Southeast, which stands out due to the amount of job opportunities. The Midwest region, in turn, has its economy focused on agriculture and stockbreeding, and presents the second lowest demographic density of the country, despite containing Brazil’s capital. The Northeast region is geographically constituted by a semiarid climate, besides presenting the lowest human development indices. The North region also presents low human development indices, occupying the second place regarding the worst levels of the country. This region is also characterized by its low demographic density, due to the vast environmental reserve constituted by the Amazon Forest, which represents an important ecosystem in the world [13].

Considering this scenario and the impact of esophageal cancer on the disease burden of the country, analysis of age-period and birth cohort as well as mortality projections are very important measures for public health planning and evaluation of effectiveness and efficacy of prevention and control actions [14–15].

In the light of the above, the main objective of the study presented herein was to analyze the effect of age, period and birth cohort regarding esophageal cancer mortality in Brazil and its geographic regions, per sex, and in 5-year age groups (after 20 years of age). Projections were also made for the period 2015–2029.

## Methods

### Study design and population

An ecological study is presented herein, which evaluates the esophageal cancer mortality rates, per sex and age group. The classification of the disease was 150 (esophagus neoplasm) and C15 (malignant esophagus neoplasm, for the 9th and 10th International Classification of Diseases, respectively), for the period 1980–2014, in age groups 0–4 and over 80 years old, for Brazil and its geographic regions, according to sex.

Mortality data were provided by the Mortality Information System (MIS), within the website of the Department of Statistics of the Brazilian Unified Health System (DATASUS) [16]. This portal provides the data in an aggregated way, whose collection and compilation is done by the Ministry of Health and made available with free access and without identification of the individuals involved. Access can be made through the website: http://www2.datasus.gov.br/DATASUS/ following the sequence: "Health Statistics (TABNET)", "Vital Statistics", "Mortality—1979 to 1995, by ICD-9" for deaths that occurred from 1980 to 1995, while the menu "Mortality—1996 to 2015, by CID -10" was accessed to obtain the deaths that occurred from 1996 to 2014. Access to such data occurred without privileged access.

Population data were obtained from the Brazilian Institute of Geography and Statistics (IBGE) [13]. For this evaluation, the population censuses of years 1980, 1991, 2000 and 2010 were utilized, also from DATASUS [17]. Data extraction on deaths and population occurred during January 15–18, 2017, and was accomplished by two different investigators.

It is important to highlight that within the study period (1980–2014), Brazil has experienced important improvements in death certification, regarding better coverage of Mortality Information Systems *(SIM)* and reduction of the proportion of ill-defined death causes [18].

### Study variables

Despite improvements in the quality of the information presented in the last decades, as shown in Table 1, there are still differences in the quality of death registries throughout the geographic regions of Brazil. A correction approach was necessary for underreporting and relocation of ill-defined death causes, based on the methodology of the World Health Organization (WHO) [19]. Deaths with incomplete diagnoses for general cancer and incomplete diagnosis for digestive tract cancer were redistributed, proportionally by year and age group [20], as described by Giusti *et al*. (2016) [21].

**Table 1:** Estimated annual percentage variation (EAPC) of esophageal cancer mortality rates, in Brazil. 1980–2014.

Sex	Region	EAPC	95% CI	
Male	North	-2.19	-2.80	-1.57
	Northeast	-4.81	-5.96	-3.65
	South	-2.96	-3.58	-2.34
	Southeast	-1.40	-1.73	-1.07
	Midweast	-4.57	-5.60	-3.52
	Brazil	-3.06	-3.72	-2.40
**Sex**	**Region**	**EAPC**	**95% CI**	
Female	North	-2.40	-3.08	-1.72
	Northeast	-4.84	-5.97	-3.7
	South	-3.06	-3.69	-2.43
	Southeast	-1.49	-1.83	-1.14
	Midweast	-4.94	-5.98	-3.88
	Brazil	-3.22	-3.89	-2.55

Esophageal cancer mortality rates were then calculated per 100,000 inhabitants, standardized by the world population as proposed by Segi (1960), for the age groups 0–4 years, 5–9 years, 10–14 years, 15–19 years, 20–24 years, 25–29 years, 30–34 years, 35–39 years, 40–44 years, 45–49 years, 50–54 years, 55–59 years, 60–64 years, 65–69 years, 70–74 years, 75–79 years, 80–84 years, and over 85 years old [22].

Analysis of the effect of age, period, and birth cohort utilized the age group from 20 years old to over 85 years old, as the younger age groups presented scarce data on deaths by the specific studied cancer, which hindered the estimation of APC models. In this way, five-year intervals were considered for the age, period, and birth cohort, yielding a total of 13 age groups, seven periods and 19 birth cohorts.

### Statistical analysis

Calculation considered Poisson Regression to evaluate the effects of age-period-cohort. It was considered that temporal effects (age-period and birth cohort) acted in a multiplicative form on the rates [14–15]. Therefore, the logarithm of the expected rate value is a linear function of the effect of age, period and cohort:

$$ln\left(E[r_{ij}]\right)=ln\left(\frac{\theta_{ij}}{N_{ij}}\right)=\mu +\alpha_{i}+\beta_{j}+\gamma_{k},$$

In this equation, *E*[*r*_*ij*_] *r*epresents the expected mortality rates for each age *i* and period *j*, *θ*_*ij*_ is the amount of deaths for each age *i* and period *j*, and *N*_*ij*_ is the population at risk of dying, for each age *i* and period *j*. Also, *μ*represents the median of the effect, while *α*_*1*_ is the effect of the age of group *i*, *β*_*j*_ is the effect of period *j*, and *γ*_*k*_ is the effect of cohort *k* [14,15]. Estimation of APC parameters presents the main limitation of not estimating the complete model, known as *non-identifiable problem*. This issue occurs due to the exact linear relationship between temporal effects (age-period and birth cohort). There is no consensus in scientific literature on the best methodology to correct this issue, and the study presented herein opted to estimate the APC effect parameters by estimable functions, proposed by Holford [14] and applied by the Epi library, from library Epi 1.1.18a. of software R version 3.2.1 (R Foundation of Computational Statistics, Vienna, Austria http://www.r-project.org).

The estimable functions limited the analysis of the effects to their linear and curve combinations. Curves are estimable functions of the parameters and remain constant independently of the parametrization applied. The linear trend of effects is divided into two components: the first is the linear effect of age and the second is the *drift*, linear effect of period and cohort [14, 15]. The longitudinal trend of age is the sum of age and slope of the period (*α*_*L*_ + *β*_*L*_),where α_L_ and *β*_*L*_ are the linear trends of age, and period, respectively. The term *drift* represents the linear trend of the logarithm of age specific rates and is equal to the sum of the slopes of period and cohort (*β*_*L*_ + *γ*_*L*_), where *β*_*L*_ and *γ*_*L*_ are the linear trends for the period and cohort, respectively [14,15].

The fit of the model was evaluated by the *deviance*, defined as twice the logarithm of the verisimilitude function of the complete model in relation to the logarithm of the verisimilitude function for the estimated model. The contribution of the effects was evaluated by comparing the *deviance* of the model with the specific effect in relation to the complete model (age-period-cohort). The results with *p*≤ 0.05 were considered statistically significant.

The reference age group was 50–54 years of age, the reference period was 1995–1999 and the reference cohort utilized a median value, as central cohorts are more stable: 1940–1944[14,15].

For each period, projections were made with the *Nordpred* [23] program within software R, using the age, period and cohort model. Data were separated in five-year blocks; however, for analysis of these data, a minimum limit of 10 cases was established for each block. Projections were separated in observed and predicted deaths, for each geographic region of Brazil and analysis period. Mortality rates were adjusted by the standard world population, for comparison purposes with published studies, expressed in 100,000 inhabitants per year. The difference between the number of deaths in the last observed period (2010–2014) and the last projected period (2025–2029) was calculated, considering the existence of variations in the proportions of changes associated with death by disease and demographic changes (size and structure of population). These components are different from zero, and present positive or negative direction. The calculation of overall total change occurred can be expressed by equation [24]:

Δtot=Δrisk+Δpop

=(Nfff−Noff)+(Noff−Nooo)

*Δtot* represents the overall total change occurred, *Δrisk* constitutes the share of change associated with the risk of developing the disease, *Δpop* is calculated on the basis of changes in size and structure of the population, *Nooo* is constituted by the number of observed deaths, while *Nfff*is the number of projected deaths and *Noff*, finally, is the number of expected deaths when there is an increase in mortality rates throughout the observed period.

## Results

For the study period, a statistically significant reduction was verified in the annual percentage change estimated for mortality due to ill-defined death certificates and those presenting codes referring to incomplete diagnosis for general cancer (195,197 to 199, 238 to 239 in CID-9 and C-76 to C-80 e C-97 in CID-10) as well as incomplete diagnosis for esophageal cancer (159 in CID-9 and C-26 in CID-10), shown in Table 1.

It must be mentioned that the study presented herein followed a correction approach necessary for relocation of ill-defined death causes and incomplete cancer diagnoses for general cancer and esophageal cancer, according to the recommendations of the WHO [19], as previously described in the study variables topic.

In the period 1980–2014, 183,602 deaths were attributed to esophageal cancer in Brazil, representing a standardized mean mortality rate of 5.40 deaths per 100,000 inhabitants. After correction for ill-defined causes there was a 7.52% increase in the number of deaths (197,305) corresponding to a mortality rate of 5.77 deaths per 100,000 inhabitants. The correction due to incomplete diagnoses for general cancer and esophageal cancer represented a 12.12% increase in the number of deaths (23,914). The correction process increased the number of deaths by 20.53% (221,219), which yields a mean mortality rate of 6.40 deaths per 100,000 inhabitants. Mortality rates were higher for men, in Brazil and its five geographic regions. In this period, adjusted mortality rates for men were higher than for women.

Evolution of esophageal cancer mortality rates in the last 35 years in Brazil suggests stationarity in mortality rates for both sexes. The rates for the last period of the historical series (2010–2014) were lower than at the beginning of the series for both sexes (1980–1984).

Regarding geographic regions, a progressive reduction was evidenced in the mortality rates for the South and Southeast regions, for both sexes. The North, Northeast and Midwest regions experienced increasing mortality rates, more pronounced for men, especially after the 2000's (Table 2).

**Table 2:** World age-standardized rates for the period 1980–2014, in Brazil, after correction of deaths.

Sex	Region	Standardized Mortality Rate	Periods						
			1980–1984	1985–1989	1990–1994	1995–1999	2000–2004	2005–2009	2010–2014
**Male**	North	ECWCR^1^	1.76	1.97	1.88	1.76	2.13	2.31	2.54
		ECCRDC^2^	2.24	2.50	2.39	2.16	2.54	2.56	2.72
		ECCRDC +GCI + DTC^3^	2.40	2.72	2.66	2.41	2.87	2.89	3.02
	Northeast	ECWCR	1.55	1.74	1.91	2.28	2.80	3.85	4.50
		ECCRDC	2.36	2.54	2.61	2.85	3.36	4.09	4.71
		ECCRDC +GCI + DTC	2.59	2.78	2.90	3.15	3.81	4.63	5.38
	Midwest	ECWCR	4.00	4.29	4.19	4.87	5.70	5.43	5.88
		ECCRDC	4.67	4.92	4.60	5.28	5.98	5.60	6.01
		ECCRDC +GCI + DTC	5.27	5.56	5.27	5.95	6.81	6.28	6.62
	Southeast	ECWCR	8.47	8.30	7.87	8.08	7.76	7.27	7.15
		ECCRDC	9.00	8.77	8.12	8.60	8.78	7.64	7.44
		ECCRDC +GCI + DTC	10.01	9.83	9.20	9.57	9.89	8.55	8.24
	South	ECWCR	13.39	13.24	12.97	12.78	12.64	10.92	10.42
		ECCRDC	14.61	14.31	13.80	13.41	13.13	11.29	10.70
		ECCRDC +GCI + DTC	15.92	15.72	15.31	15.06	14.45	12.45	11.85
	Brazil	ECWCR	6.73	6.71	6.00	6.21	6.77	6.61	6.63
		ECCRDC	7.47	7.39	7.03	7.25	7.51	6.93	6.87
		ECCRDC +GCI + DTC	8.24	8.20	7.89	8.09	8.42	7.74	7.65
**Female**	North	ECWCR	0.48	0.49	0.52	0.54	0.58	0.63	0.74
		ECCRDC	0.59	0.60	0.65	0.66	0.68	0.68	0.79
		ECCRDC +GCI + DTC	0.63	0.68	0.75	0.73	0.79	0.78	0.89
	Northeast	ECWCR	0.47	0.59	0.55	0.71	0.92	1.24	1.30
		ECCRDC	0.69	0.84	0.75	0.91	1.10	1.31	1.49
		ECCRDC +GCI + DTC	0.77	0.93	0.85	1.02	1.26	1.53	1.69
	Midwest	ECWCR	1.04	1.26	1.22	1.75	1.52	1.48	1.40
		ECCRDC)	1.18	1.41	1.32	1.87	1.58	1.51	1.42
		ECCRDC +GCI + DTC	1.25	1.56	1.63	2.17	1.85	1.74	1.84
	Southeast	ECWCR	2.21	2.10	1.81	1.80	1.61	1.48	1.38
		ECCRDC	2.34	2.20	1.91	1.90	1.70	1.54	1.42
		ECCRDC +GCI + DTC	2.69	2.57	2.24	2.19	1.94	1.68	1.64
	South	ECWCR	3.88	3.64	3.43	3.36	3.17	2.68	2.46
		ECCRDC	4.24	3.92	3.64	3.60	3.27	2.76	2.51
		ECCRDC +GCI + DTC	4.84	4.46	4.19	3.88	3.68	3.12	2.84
	Brazil	ECWCR	1.86	1.81	1.64	1.41	1.30	1.57	1.50
		ECCRDC	2.05	1.99	1.70	1.54	1.39	1.64	1.58
		ECCRDC +GCI + DTC	2.34	2.29	2.08	2.07	1.98	1.84	1.81

^1^Esophageal cancer without correction (ECWCR)

^2^Esophageal cancer correction for ill-defined causes (ECCRDC)

^3^Esophageal cancer correction for ill-defined causes and diagnoses incomplete general cancer and cancer of the digestive tract (ECCRDC +GCI + DTC)

**Fig 1:**
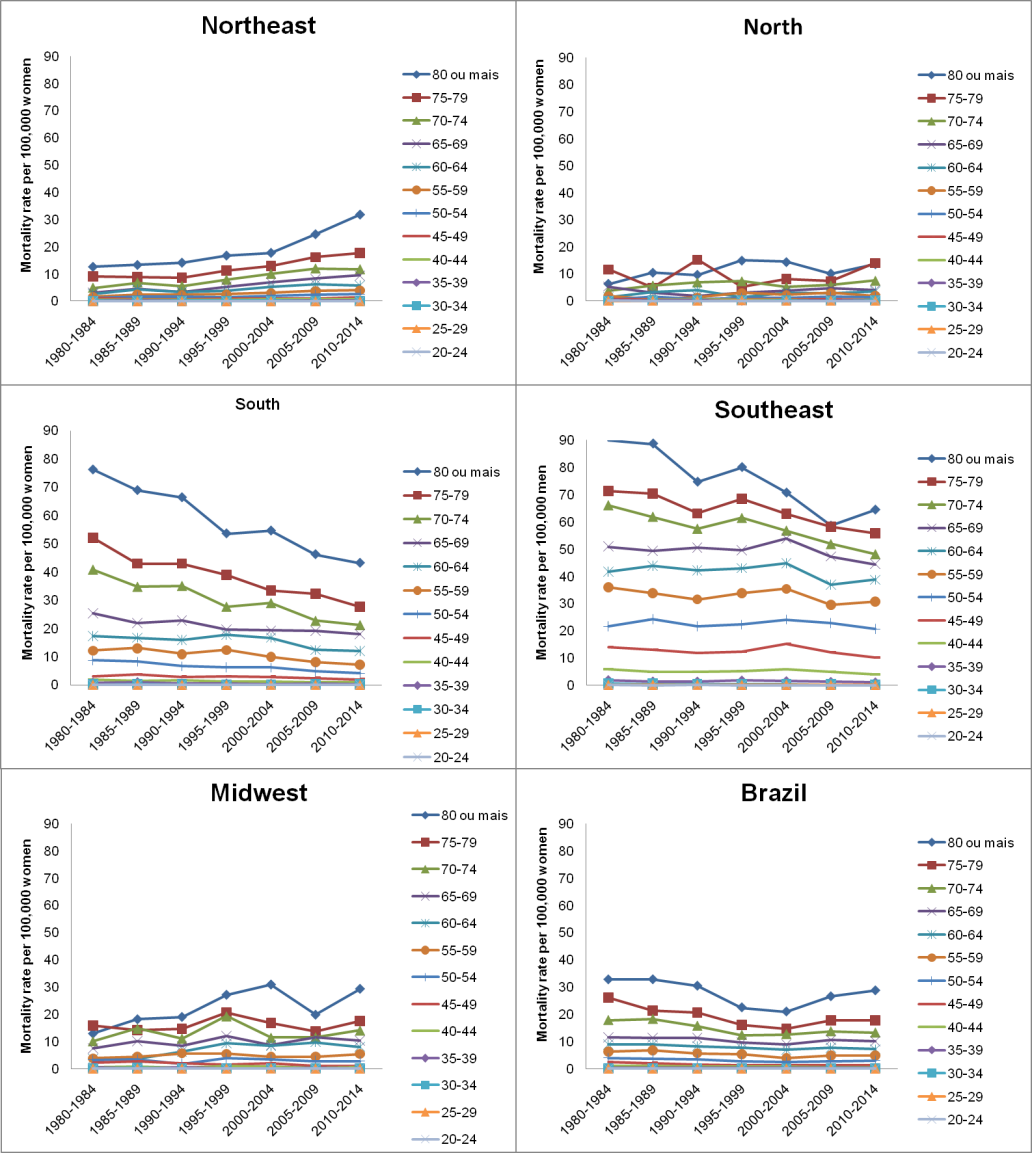
Mortality rates for esophageal cancer in females, according to age, death period, and geographical region in Brazil.

**Fig 2:**
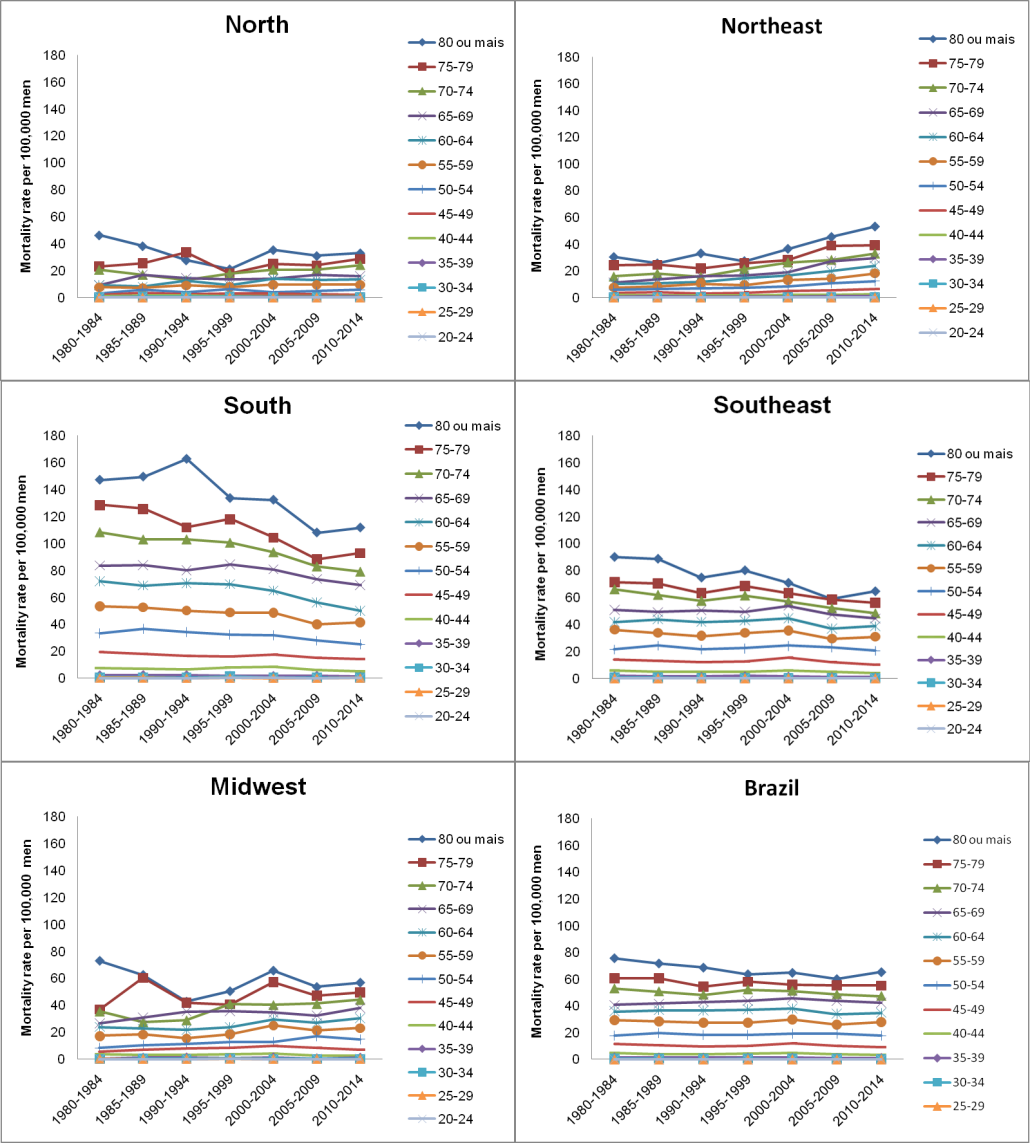
Mortality rates for esophageal cancer in males, according to age, death period, and geographical region in Brazil.

In Brazil and its five geographic regions, and in both sexes, the exploratory analysis of esophageal cancer mortality rates revealed a progressive increase after the age group 60–64 years old. The highest rates were verified for the age group over 80 years old (Figs 1 and 2). This profile is maintained when the effect of age is adjusted by the effect of the period and birth cohort, when estimating the complete model.

Regarding mortality rates per age groups, according to the birth cohorts, there was a decreasing pattern in the evolution of mortality rates after the 1950's, for men and women in Brazil, as well as for the South, Southeast and Midwest regions. For the North region, an increase was detected in mortality rates for men and women, in more advanced age groups, starting from the 1920 birth cohort. Regarding the Northeast region, a similar pattern was identified, for both sexes, for individuals born after the 1910–1914 cohort, for individuals over 65–69 years of age. For the Midwest region, after the 1960's and for age groups over 60 years, increasing mortality rates were also detected (S1 and S2 Appendices).

**Fig 3:**
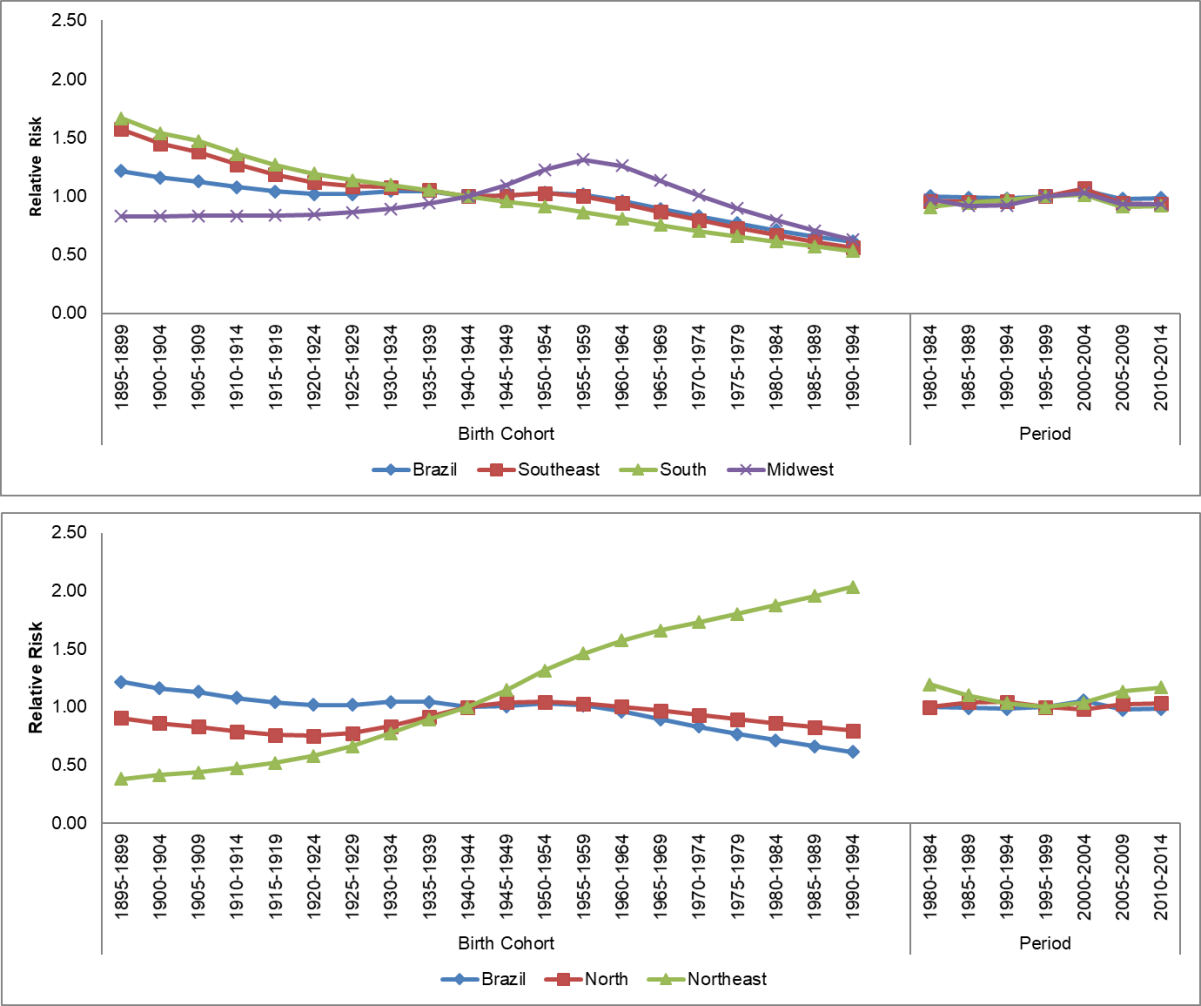
Results of the age-period-cohort model, adjusted for esophageal cancer mortality in males, according to geographical region, in Brazil.

**Fig 4:**
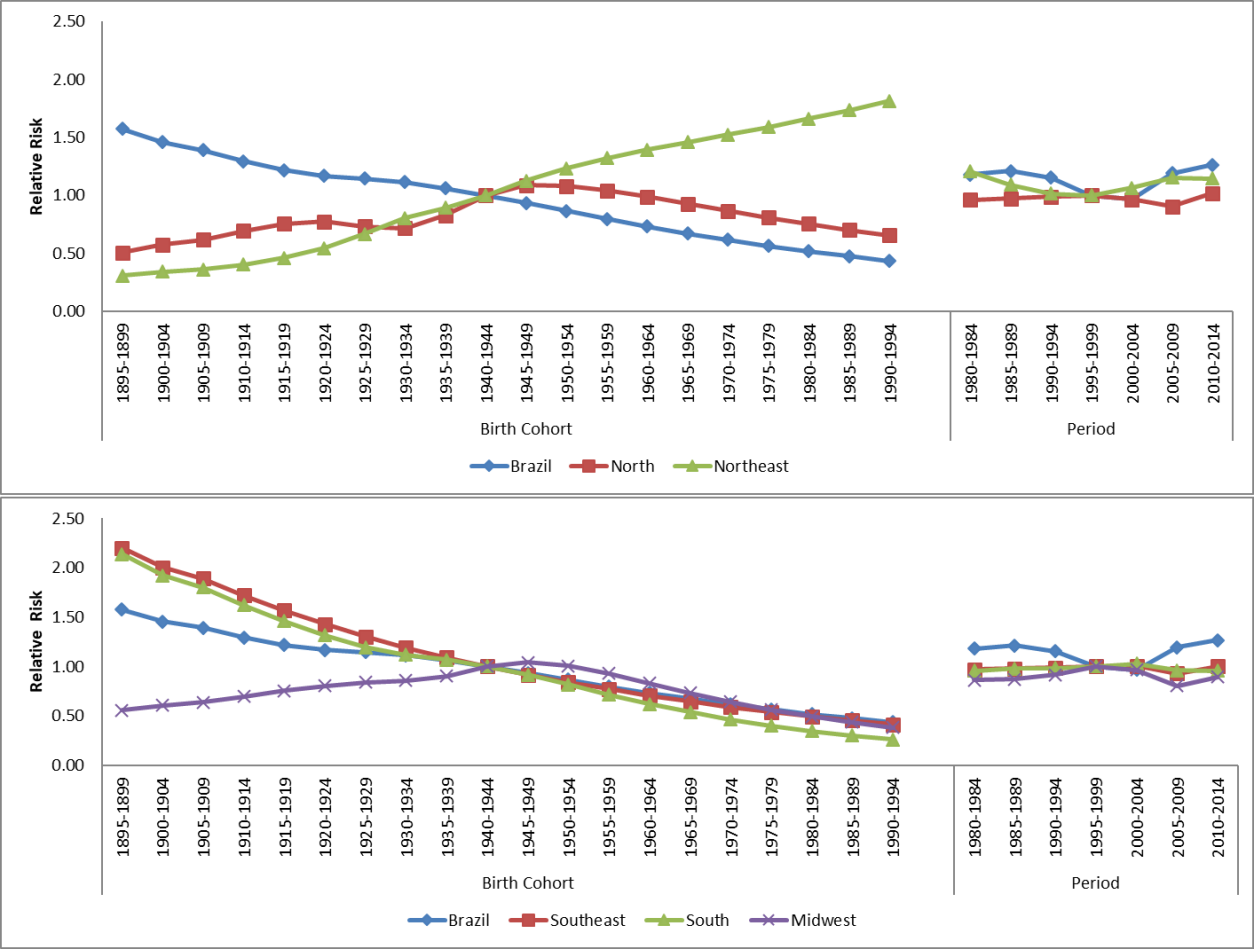
Results of the age-period-cohort model, adjusted for esophageal cancer mortality in females, according to geographical region, in Brazil.

Concerning death risk for the analyzed periods, there was a protection effect for Brazil (with relative risk, RR, under 1) for the last periods of the series (2005–2009) and 2010–2014) in relation to the reference period (1995–1999) for men. For women, an increasing death risk was present for the last periods of the historical series. Analysis of death risk per geographic region points to disparities across Brazilian regions. For the North and Northeast regions, there was an increase in risk (RR>1) in relation to the reference period in the two last periods analyzed (Fig 3). The Southeast, South, and Midwest regions showed decreasing death risk for this neoplasm, with a protection effect (RR<1), especially in periods 2005–2009 and 2010–2014 for both sexes (Fig 4).

Similarly, there were disparities in death risks for the different Brazilian geographic regions. For Brazil and both sexes, as well as for the South and Southeast regions, there was a progressive reduction in death risk after 1940's. For the Midwest region, there was an increase in death risk after the 1945–1949 cohort until 1950–1955, and then a progressive reduction was observed for the birth cohorts after. A similar profile was observed for the Midwest region, with an increase in death risk after the 1945–1949 cohort until 1950–1955, and then a progressive reduction was observed for the birth cohorts after this. The Northeast region, nevertheless. presented a progressive increase in death risk for men and women (RR>1) after the 1945–1949 cohort (Fig 1).

Table 3 shows the deviance changes in the sequential construction of APC models. Regarding the evolution of rates for men and women, in Brazil and its geographic regions, the best adjusted model according to the value of deviance and p-value at the level of 5%, was the APC model. The South region for women and North region for men were exceptions, as in these cases the most explicative model was age-cohort (AC)—and the North region for women—the best-adjusted model was age-drift.

**Table 3:** Deviance changes in the sequential construction of APC models.

Brazil						
	Female			Male		
Models	DF^1^	Resid DV^2^	Pr(>Chi)	DF	Resid DV	Pr(>Chi)
Age	85	1,114.57		85	1,098.9	
Age-drift^3^	84	741.15	<0.0001	84	1,012.46	<0.0001
Age-Cohort	80	685.32	<0.0001	80	836.16	<0.0001
Age-Period-Cohort	77	221.98	<0.0001	77	731.86	<0.0001
Age-Period	81	269.71	<0.0001	81	889.7	<0.0001
Age-drift^4^	84	741.15	<0.0001	84	1,012.46	<0.0001
Midwest						
Models	DF	Resid DV	Pr(>Chi)	DF	Resid DV	Pr(>Chi)
Age	85	133.524		85	234.04	
Age-drift	84	131.143	0.123	84	189.87	<0.0001
Age-Cohort	80	108.197	0.0001	80	149.45	<0.0001
Age-Period-Cohort	77	96.675	0.009	77	136.23	<0.0001
Age-Period	81	116.76	0.0004	81	174.94	<0.0001
Age-drift	84	131.143	0.002	84	189.87	0.002
North						
Models	DF	Resid DV	Pr(>Chi)	DF	Resid DV	Pr(>Chi)
Age	85	126.41		85	136.86	
Age-drift	84	120	0.0114	84	124.94	5E-04
Age-Cohort	80	111.75	0.0831	80	109.75	0.004
Age-Period-Cohort	77	110.04	0.6336	77	108.29	0.691
Age-Period	81	118.41	0.078	81	123.78	0.003
Age-drift	84	120	0.6631	84	124.94	0.764
Northeast						
Models	DF	Resid DV	Pr(>Chi)	DF	Resid DV	Pr(>Chi)
Age	85	827.08		85	1,667.97	
Age-drift	84	192.82	<0.0001	84	247.88	<0.0001
Age-Cohort	80	157.45	<0.0001	80	214.79	<0.0001
Age-Period-Cohort	77	135.23	<0.0001	77	151.34	<0.0001
Age-Period	81	171.39	<0.0001	81	182.38	<0.0001
Age-drift	84	192.82	<0.0001	84	247.88	<0.0001
South						
Models	DF	Resid DV	Pr(>Chi)	DF	Resid DV	Pr(>Chi)
Age	85	610.52		85	987.83	
Age-drift	84	142.65	<0.0001	84	458.72	<0.0001
Age-Cohort	80	120.64	0.0001	80	443.65	0.004
Age-Period-Cohort	77	111.62	0,0643	77	365.2	<0.0001
Age-Period	81	132.12	0.0034	81	375.99	0.029
Age-drift	84	142.65	0.032	84	458.72	<0.0001
Southeast						
Models	DF	Resid DV	Pr(>Chi)	DF	Resid DV	Pr(>Chi)
Age	85	927.12		85	1,135.61	
Age-drift	84	162.93	<0.0001	84	819.37	<0.0001
Age-Cohort	80	162.6	0.9883	80	701.07	<0.0001
Age-Period-Cohort	77	144.5	0.0117	77	523.39	<0.0001
Age-Period	81	144.99	0.974	81	639.7	<0.0001
Age-drift	84	162.93	0.0112	84	819.37	<0.0001

^1^ Degrees of freedom

^2^ Residual Deviance

^3^ linear trend of the logarithm of age specific rates, and is equal to the sum of the slopes of period and cohort (β_L_ + γ_L_), where β_L_ and *γ*_*L*_ are the linear trends for the period and cohort.

^4^ longitudinal trend of age is the sum of age and slope of the period (α_L_ + β_L_), where α_L_ and *β*_*L*_ are the linear trends of age, and period, respectively

Projections of standardized mortality rates for esophageal cancer show increases for men in the Northeast region, and for women in the North and Northeast regions. Projections for the Midwest, South and Southeast regions as well as the pooled analysis for Brazil indicated a reduction in mortality rates for both sexes. Projections per sex are presented in Tables 4 and 5, with the adjusted rates for the observed period and the number of cases per region.

**Table 4:** Observed and predicted number of deaths in males by age and world age-standardized rates (ASW) in Brazil.

	Observed			Predicted		
	2000–2004	2005–2009	2010–2014	2015–2019	2010–2024	2025–2029
**BRAZIL**						
**Age (years)**						
0–49	4,424	4,411	4,215	3,910	3,753	4,389
50–74	18,647	21,141	24,038	27,241	24,908	31,555
≥ 75	4,656	5,880	6,799	6,965	6,535	9,845
ASW	8.42	7.74	7.65	6.89	6.37	6.00
95% CI	8.23–8.51	7.62–7.88	7.51–7.73	6.79–6.91	6.21–6.43	5.92–6.03
**Northeast**						
**Age (years)**						
0–49	451	586	790	926	1,052	1,081
50–74	2,115	2,943	4,075	5,401	6,745	8,017
≥ 75	833	1,219	1,539	1,688	2,170	2,864
ASW	3.81	4.63	5.38	6.06	6.46	6.63
95% CI	3.61–3.94	4.56–4.84	5.25–5.63	5.93–6.21	6.31–6.57	6.47–6.80
**North**						
**Age (years)**						
0–49	77	92	95	117	134	150
50–74	340	449	564	688	830	985
≥ 75	122	148	196	204	257	324
ASW	2.87	3.01	3.02	3.01	2.98	2.92
95% CI	2.61–3.13	2.79–3.29	2.87–3.34	2.81–3.23	2.79–3.15	2.74–3.09
**Midwest**						
**Age (years)**						
0–49	252	233	256	261	324	347
50–74	889	1,126	1,427	1,767	2,013	2,364
≥ 75	246	294	361	405	534	699
ASW	6.81	6.28	6.62	6.29	6.09	6.00
95% CI	6.55–6.98	6.15–6.57	6.36–6.87	6.01–6.57	5.82–6.31	5.76–6.20
**Southeast**						
**Age (years)**						
0–49	2,558	2,434	2,080	1,818	1,775	1,758
50–74	10,102	10,957	12,000	13,190	13,853	14,536
≥ 75	2,178	2,710	3,020	3,047	3,384	4,106
ASW	9.89	8.55	8.24	7.03	6.31	5.84
95% CI	9.59–9.97	8.42–873	7.95–8.46	6.92–7.15	6.22–6.36	5.76–5.95
**South**						
**Age (years)**						
0–49	1,085	1,067	992	813	839	976
50–74	5,204	5,666	5,971	6,590	6,853	7,176
≥ 75	1,278	1,510	1,683	1,722	1,964	2,327
ASW	14.45	12.45	11.85	9.96	8.98	8.49
95% CI	14.21–14.62	12.14–12.63	11.63–12.05	9.71–10.17	8.76–9.15	8.27–8.65

**Table 5:** Observed and predicted number of deaths in females by age and world age-standardized rates (ASW) in Brazil.

	Observed			Predicted		
	2000–2004	2005–2009	2010–2014	2015–2019	2010–2024	2025–2029
**BRAZIL**						
**Age (years)**						
0–49	753	772	819	815	914	1,092
50–74	4,645	5,330	5,865	6,705	7,433	8,121
≥ 75	2,578	3,217	3,941	4,195	4,783	5,700
ASW	1.98	1.84	1.81	1.65	1.55	1.49
95% CI	1.81–2.03	1.78–1.95	1.75–1.93	1.54–1.74	1.46–1.61	1.41–1.56
**Northeast**						
**Age (years)**						
0–49	132	154	218	213	265	308
50–74	767	1,079	1,275	1,613	1,847	2,118
≥ 75	492	762	1,132	1,301	1,595	1,920
ASW	1.26	1.53	1.69	1.74	1.73	1.73
95% CI	1.15–1.35	1.44–1.61	1.58–1.74	1.67–1.79	1.67–1.79	1.66–1.79
**North**						
**Age (years)**						
0–49	21	16	29	27	31	35
50–74	79	121	145	192	246	296
≥ 75	53	51	105	143	207	280
ASW	0.79	0.78	0.89	1.01	1.06	1.07
95% CI	0.57–0.92	0.58–0.93	0.65–1.03	0.92–1.18	0.98–1.18	0.98–1.19
**Midwest**						
**Age (years)**						
0–49	55	42	45	49	54	57
50–74	220	319	384	494	574	669
≥ 75	109	115	190	241	343	475
ASW	1.85	1.74	1.84	1.78	1.71	1.67
95% CI	1.71–2.01	1.58–1.95	1.70–2.01	1.61–1.95	1.59–1.84	1.43–1.82
**Southeast**						
**Age (years)**						
0–49	362	363	358	347	383	393
50–74	2,147	2,333	2,546	2,924	3,284	3,682
≥ 75	1,200	1,374	1,589	1,612	1,766	2,078
ASW	1.94	1.68	1.64	1.47	1.4	1.37
95% CI	1.85–2.01	1.78–1.79	1.55–1.75	1.38–1.54	1.32–1.43	1.30–1.43
**South**						
**Age (years)**						
0–49	188	197	169	146	119	109
50–74	1,433	1,478	1,518	1,533	1,584	1,634
≥ 75	725	914	924	950	1,026	1,223
ASW	3.68	3.12	2.84	2.25	1.95	1.78
95% CI	3.54–3.81	3.06–3.32	2.65–3.01	2.16–2.37	1.87–2.05	1.71–1.84

**Fig 5:**
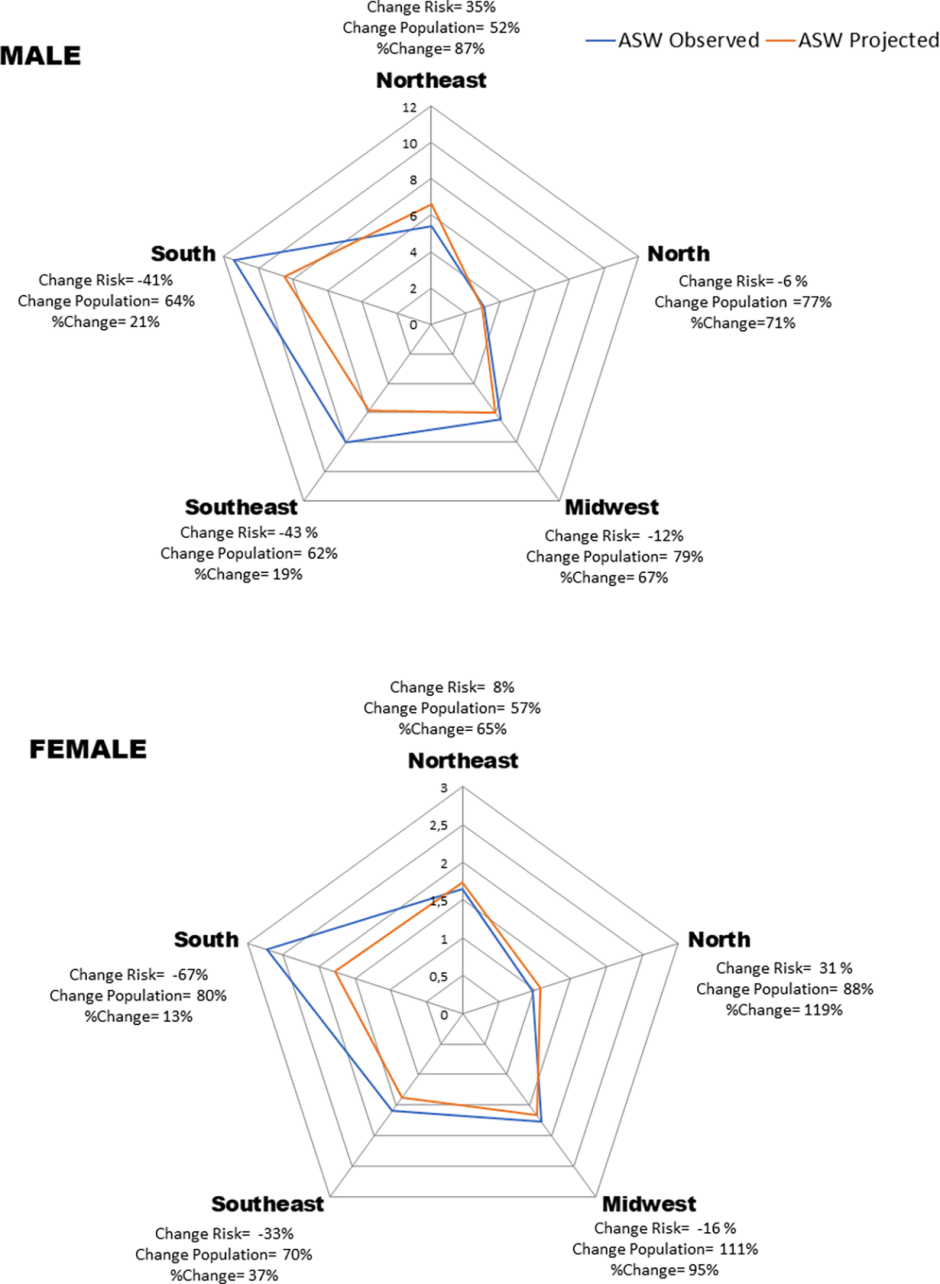
World age-standardized rates (ASW), changes in numbers of deaths (No) relative change due to risk (Risk) and changes in the structure of population (Pop), between 2010–2014 (observed) and 2025–2029 (predicted) of esophageal cancer mortality in Brazil.

An increase is expected in the number of deaths (10,737) for the male population in Brazil, when comparing the last observed period in relation to the last projected period, representing a 30% growth, of which 62% is due to population increase and -32% is due to reduction in risk. Regarding women, the expected increase in the number of deaths was 4,288, with 40% growth due to changes in population and -32% in reduction of risk and 72% to population increase. The modifications in the number of deaths, separated by sex, and in function of the risk of developing esophageal cancer and changes in population structure, are represented in Fig 5, which compares the last observed period with the last projected period for Brazil and its geographic regions.

## Discussion

Brazil is a country of continental dimensions, with pronounced socioeconomic disparities and healthcare inequalities, which could explain the differences in the demographic and epidemiologic transition processes experienced by its five geographic regions [25–26].

While the South and Southeast regions present the best socioeconomic indicators, the North and Northeast regions suffer with the worst indicators. This reality strongly affects the access to health services, even though Brazil counts with a public universal healthcare system [25–26].

Herein the highest mortality rates were verified for the South and Southeast regions, with values similar to the rates observed in countries of Oriental Asia and South of Africa [1–3]. The lowest mortality rates were verified for the North and Northeast regions of Brazil [1–3]. The Northeast region presented an intermediate level, with rates similar to those of Uruguay, Argentina and Chile [5]. The North region presented mortality rates equivalent to those of United Kingdom, Germany and Cuba [27].

The high mortality rates for esophageal cancer in the Brazilian regions with higher socioeconomic development can be explained by the heterogeneous demographic process of the country. According to Vasconcelos & Gomes (2012) [28], since the 20th century there have been drops in mortality, birth rates and fecundity in Brazil, which started the aging process of the population. These changes, however, did not occur concomitantly nor homogeneously throughout the country. While the Southeast, South and Midwest regions present a more advanced process, the North and Northeast regions present younger age structure, as these regions still present high fecundity, birth and mortality rates.

In this context, it is important to highlight that the industrialization and urbanization process, along with changes in habits and lifestyle, has been more intense in the South and Southeast regions of Brazil. This way, the epidemiological transition was more pronounced, with a progressive increase in incidence and mortality rates of chronic non-transmissible diseases, among these, cancer. The North and Northeast regions also present epidemiological transition processes, but these are characterized by superposition rather than substitution, with high morbidity burden due to transmissible diseases and external causes [28–29]. The cancer transition defended by Bray et al (2012) [30] was observed herein, where locations with lower human development indices are more exposed to cancers associated with infections (cervical, liver, non-Hodgkin lymphoma, among others) while regions with higher human development indices are associated with lifestyle and habit-related cancers. Higher prevalence of tobacco smoking must also be mentioned, which was an important risk factor for esophageal cancer in the South and Southeast regions, when compared with the North and Northeast [31, 32].

The South region of Brazil, despite presenting the highest socioeconomic development and best healthcare indicators, is recognizably the Brazilian location with the highest incidence and mortality rates for esophageal cancer, and this situation has been maintained for decades. The mortality pattern of the Midwest region has been the same for the last two analyzed periods, and presented higher rates in comparison with the 1980's, which were similar to those of countries with intermediate mortality.

This reality is a reflex of the particularities of each region, with habits and lifestyle connected to local culture. In South Brazil, as well as in Argentina and Uruguay, yerba mate tea (*chimarrão*) is consumed at high temperatures, several times a day [33]. In this sense, it is important to remark that studies have demonstrated an association between the consumption of yerba mate tea and esophageal cancer, based on meta-analysis studies [34] and pooled analyses [35]. These studies evidenced a positive association with the high consumption of yerba mate tea, when compared with low consumption. These case-control studies evaluated by pooled analyses were adjusted by the consumption of tobacco and alcohol, as well as by sex, age group, education level, and housing location (urban/rural) [35].

The results observed for the Midwest region can be explained by the intense migration of southerners to this region in the 1970's, due to the expansion of agriculture frontiers. Migrants maintained the same cultural habits of drinking yerba mate tea, and a study carried out by Silva et al (2013) [36] calculated the incidence rate of esophageal cancer in two capitals of the Midwest: Cuiabá (Mato Grosso state) and Brasília (Federal District), which were similar to the incidence rates observed for the South region.

The South and Southeast regions were the first Brazilian regions to count with sanitation and access to consumption goods, such as refrigerators. This could explain the reduction in mortality rates by gastric cancer presented in the last 30 years, and the increase in mortality rates of the North and Northeast regions, in the same period, especially after the 2000’s [21]. It is possible that decreasing mortality rates for gastric cancer are a consequence of decreasing prevalence of *Helicobacter pylori*; such a decrease can reduce gastric cancer incidence and mortality and simultaneously contribute to increase esophageal cancer. The stomach infected by this bacterium produces less acid and inhibits the synthesis of ghrelin, which induces satiety—ghrelin prevents obesity and promotes gastric emptying. All these associated elements decrease the possibility of gastroesophageal reflux, a known risk factor, especially for esophageal adenocarcinoma [37–39].

It was also observed, for both sexes and for all geographic regions, a progressive increase in mortality rates with the aging process, reaching an incidence peak in individuals in their 60's. These results corroborate those found in Korea [40], Osaka-Japan [41], Linzhou-China [42], Australia [43], Spain [44], Germany [27], Cuba [27] and the United Kingdom [27]. This was expected, as there is strong effect of age on the evolution of incidence and mortality rates of chronic diseases, which are a consequence of exposure to risk factors throughout life [14–15].

The similarity between esophageal cancer incidence and mortality rates must be mentioned, due to poor prognosis, as frequently diagnosis is made at advanced stages of the disease. Dysphagia, the main symptom of the disease, only manifests when a considerable extension of the organ is compromised (generally the distal two-thirds of the esophagus) [45–46]. Associated with late diagnosis, prognosis for this neoplasm is still reserved, as no improvements in survival were observed with adjuvant chemotherapy [47]. However, recent clinical advances have evidenced therapeutic improvements regarding neoadjuvancy implemented with the association of chemoradiotherapy for resectable tumors [43, 48–49].

The differences observed in esophageal cancer mortality for men and women, across Brazilian geographic regions, agree with other studies [50, 40–44]. Men present higher incidence and mortality due to higher exposure to risk factors for this neoplasm [9–12], and because men utilize less health services, such as medical tests and appointments [50].

Regarding death risk in the 5-year periods analyzed and projections, the male sex and regions South, Southeast and Midwest presented decreases in the last two 5-year periods and when comparing the last observed period with the last projected period. These results are similar to those observed in Chile [5], Costa Rica [5], Argentina [5], Japan [41], Germany [27] and Spain [44]. This result could be a reflex of the interaction between the effect of healthcare service access period and birth cohort, reducing risk factor prevalence for esophageal cancer in the population of those regions (especially regarding tobacco consumption) [5–14,19–21,25–27,41–44].

The period effect refers to alterations in incidence and mortality trends, related to the changes that occur in a specific period and influence simultaneously all age groups [14–15]. Therefore, possibly the increase in death risk presented by regions North and Northeast, which remained in the projections, can be a consequence of improvements in *SIM* and death registries. This could have reduced the proportion of ill-defined registered deaths due to better access to health services and cancer assistance, after implementation of the National Policy for Cancer Attention [51]. The possibility of diagnosing the disease was increased, even if in late stages.

The birth cohort effect is caused by factors that affect one generation, promoting changes with different magnitudes in successive age and period groups, and enables the analysis of long-term exposure effects to risk factors. Still, the effect of birth cohort reflects the interaction between the effect of age and period as a result of accumulated exposure throughout time [14–15].

In this study, the progressive reduction in esophageal cancer death risk for birth cohorts after the 1950's is remarkable for Brazil, the South, Southeast and North regions, for both sexes, and after the 1960's for the Midwest region. This decrease in death risk can be a consequence of the birth cohort effect promoted by the reduction of tobacco consumption prevalence in these populations [40–44]. This hypothesis is reinforced because there has been a decrease in death risk, in Brazil, for esophageal [21] and lung [52] cancers starting with the same cohorts. Brazil has presented an important reduction in tobacco consumption prevalence in the last two decades, related to prevention and control measures that include education, prevention, treatment and policy actions [31–32, 45].

In contrast, there was an increase in death risk in the 1940's for both sexes in the Northeast region. It is possible that, in this region, the poorest of Brazil, this reality could be explained by the interaction between the period and birth cohort effects, due to higher possibilities of diagnosing cancer along with better access to health services. Increased exposure to risk factors, such as tobacco consumption, is not probable, as studies have demonstrated reductions in tobacco prevalence for this geographic region, between 1989 and 2013 [31–32, 45].

The impossibility of evaluating the dynamics of mortality rates according to histological type is a limitation of the study. Esophageal spinocellular carcinoma and adenocarcinoma present different risk and protection factors [9–12] and according to recent studies, present different behavior regarding incidence and mortality [8]. The Brazilian Mortality Information System does not provide information on the histological type, one of its fragilities in the evaluation of the profile of cancer mortality in the Brazilian population. However, a study carried out with incidence data from population-based cancer registries in Brazil has identified that more than 70% of incident cases refer to squamous cell carcinoma [5].

Another vulnerability related to the Mortality Information System refers to the intra-regional differences in death registry quality. Nevertheless, this study attenuated this limitation by correcting the ill-defined death registries and those with incomplete diagnoses, through proportional distribution [19–21]. Additionally, Brazil does not count with a historical series on the prevalence of risk and protection factors for non-transmissible chronic diseases, which could certainly help better comprehend the behavior of mortality rates in different birth cohorts.

It must also be highlighted that there are inherent fragilities of the APC method. However, this type of study is utilized since the 1980's, although scientific literature does not reach a consensus on the best statistical method for the correction of unidentified problems [14].

## Supporting information

S1 Appendix Mortality rates for esophageal cancer by birth cohort and age in females, according geographical region, in Brazil. (TIF)

S2 Appendix Mortality rates for esophageal cancer by birth cohort and age in males, according to geographical region, in Brazil. (TIF)

## References

[1] Ferlay J, Soerjomataram I, Ervik M, Dikshit R, Eser S, Mathers C, et al GLOBOCAN 2012 v1.0, Cancer incidence and mortality worldwide: IARC Cancer Base No. 11 International Agency for Research on Cancer 2014 Available from: http://globocan.iarc.fr.

[2] Thuler FP, Forones NM, Ferrari AP. Advanced esophageal cancer: still a delayed diagnosis. Arq Gastroenterol. 2006; 43(3):206–11. 17160236 10.1590/s0004-28032006000300010

[3] American Cancer Society. Global cancer facts & figures 2008 [Internet]. 2nd ed. Atalanta: American Cancer Society; 2011 Available from: http://www.cancer.org/acs/groups/content/@epidemiologysurveilance/documents/document/acspc-027766.pdf.

[4] Thrift AP, Whiteman DC. The incidence of esophageal adenocarcinoma continues to rise: analysis of period and birth cohort effects on recent trends. Annals of Oncology 23: 3155–3162, 2012 doi: 10.1093/annonc/mds181 22847812 10.1093/annonc/mds181

[5] Barrios E, Sierra MS, Musseti C, Forman D. The burden of oesophageal cancer in Central and South America. Cancer Epidemiology 44S (2016) S53–S61. 10.1016/j.canep.2016.03.013 27678323

[6] Simard EP, Ward EM, Siegel R, Jemal A. Cancers with increasing incidence trends in the United States: 1999 Through 2008. Ca Cancer J Clin, 2012; 62:118–128. doi: 10.3322/caac.20141 22281605 10.3322/caac.20141

[7] Stewart B., Wild C. (Eds.). Oesophageal cancer In: World cancer report 2014. Lyon, France: International Agency for Research on Cancer, 2014.

[8] Arnold M, Soerjomataram I, Ferlay J, Forman D. Global incidence of oesophageal cancer by histological subtype in 2012. Gut 2015;64:381–387. doi: 10.1136/gutjnl-2014-308124 25320104 10.1136/gutjnl-2014-308124

[9] Edgren G, Adami HO, Weiderpass E, Nyrén O. A global assessment of the oesophageal adenocarcinoma epidemic. Gut 2013; 62:1406–1414. doi: 10.1136/gutjnl-2012-302412 22917659 10.1136/gutjnl-2012-302412

[10] Holmes RS, Vaughan TL. Epidemiology and pathogenesis of esophageal cancer. Semin Radiat Oncol. 2007 1;17(1):2–9. doi: 10.1016/j.semradonc.2006.09.003 17185192 10.1016/j.semradonc.2006.09.003

[11] Azevedo e Silva G, Moura L, Curado MP, Gomes FS, Otero U, Rezende LFM. The Fraction of Cancer Attributable to Ways of Life, Infections, Occupation, and Environmental Agents in Brazil in 2020.PLoS One. 2016; 11(2): e0148761 doi: 10.1371/journal.pone.0148761 26863517 10.1371/journal.pone.0148761 PMC4749327

[12] Olsen CM, Pandeya N, Green AC, Webb PM, David C. Population Attributable Fractions of Adenocarcinoma of the Esophagus and Gastroesophageal Junction. Am J Epidemiol. 2011;174(5):582–590. doi: 10.1093/aje/kwr117 21719746 10.1093/aje/kwr117

[13] Brazilian Institute of Geography and Statistics. Available from: http://www.ibge.gov.br/home/estatistica/populacao/estimativa2016/estimativa_dou.shtm.

[14] Holford TR. Understanding the effects of age, period, and cohort on incidence and mortality rates. Annu Rev Public Health 1991; 12: 425–457 [doi: 10.1146/annurev.pu.12.050191.002233 ]. 2049144 10.1146/annurev.pu.12.050191.002233

[15] Robertson C, Gandini S, Boyle P. Age-period-cohort models: a comparative study of available methodologies. J Clin Epidemiol 1999; 52: 569–583 [doi: 10.1016/ S0895-4356(99)00033-5 ]. 10408997 10.1016/s0895-4356(99)00033-5

[16] Computer science department of SUS. SIM—Mortality Information System. Available from: <http://datasus.saude.gov.br/sistemas-e-aplicativos/eventos-v/sim-sistema-de-informacoes-de-mortalidade>. Acessed in: january 16, 2016.

[17] Computer science department of SUS (DATASUS). Resident population. Available from:http://tabnet.datasus.gov.br/cgi/deftohtm.exe?ibge/cnv/popbr.def.

[18] Brasil. Ministério da Saúde. Secretaria de Vigilância em Saúde. Departamento de Vigilância de Doenças e Agravos Não Transmissíveis e Promoção da Saúde. Saúde Brasil 2014: uma análise da situação de saúde e das causas externas / Ministério da Saúde, Secretaria de Vigilância em Saúde, Departamento de Vigilância de Doenças e Agravos Não Transmissíveis e Promoção da Saúde.–Brasília: Ministério da Saúde, 2015. 462 p.: il.

[19] Mathers CD, Bernard C, Iburg KM, Inoue M, Fat DM, Shibuya K, Stein C, Tomijima N, Xu H. Global Burden of Disease in 2002: data sources, methods and results. Global Programme on Evidence for Health Policy Discussion Paper No. 54 World Health Organization December 2003 (revised February 2004).

[20] Mello JMH.; Gotlieb SLD; Laurenti R. O sistema de informações sobre mortalidade: problemas e propostas para o seu enfrentamento I-mortes por causas naturais.Rev. bras. Epidemiol. 2002; 5(2):197–211.

[21] Giusti ACB, Salvador PTCO, Santos J, Meira KC, Camacho AR, Guimarães RM, Souza DLB. Trends and predictions for gastric cancer mortality in Brazil. World J Gastroenterol 2016 7 28; 22(28): 6527–6538. doi: 10.3748/wjg.v22.i28.6527 27605887 10.3748/wjg.v22.i28.6527 PMC4968132

[22] Segi M. Cancer mortality for selected sites in 24 countries (1950–57) Department of Public Health, Tohoku University of Medicine, Sendai, Japan 1960.

[23] Nordpred Software Package. Available from: https://www.kreftregisteret.no/en/Research/Projects/Nordpred/Nordpred-software/.

[24] Møller B1, Fekjaer H, Hakulinen T, Sigvaldason H, Storm HH, Talbäck M, Haldorsen T. Prediction of câncer incidence in the nordic countries: Empirical comparison of diferent approaches. Stat Med. 2003; 22:2751–2766. doi: 10.1002/sim.1481 12939784 10.1002/sim.1481

[25] Omran A. The epidemiologic transition: a theory of the epidemiology of population change. Milbank Memorial Fund Quarterly, 49 (Part 1), p. 509–538, 1971. 5155251

[26] Malta DC, da Silva JB Jr. Plano de Ações Estratégicas para o Enfrentamento das Doenças Crônicas Não Transmissíveis no Brasil após três anos de implantação, 2011–2013. Epidemiol. Serv. Saúde, Brasília, v. 23, n. 3, p.599–608, Set. 2014.

[27] World Health Organization. WHO mortality data and statistics [Internet]. Geneva: WHO; 2013 Available from: http://www.who.int/whosis/mort/download/en/index.html.

[28] Vasconcelos AMN, Gomes MMF. Transição demográfica: a experiência brasileira. Epidemiol. Serv. Saúde[Internet]. 2012 21(4): 539–548. http://dx.doi.org/10.5123/S1679-49742012000400003.

[29] Guimarães RM, Muzi CD, Teixeira MP, Pinheiro SS. A transição da mortalidade por cânceres no Brasil e a tomada de decisão estratégica nas políticas públicas de saúde da mulher. R. Pol. Públ., São Luís, v. 20, n 1, p. 33–50, Jan-Jun 2016.

[30] Bray F, Jemal A, Grey N, Ferlay J, Forman D. Global cancer transitions according to the Human Development Index (2008–2030): a population-based study. Lancet, [S. l.], v. 13, n. 8, p.1–11, 8 2012. 10.1016/S1470-2045(12)70211-5 22658655

[31] Malta DC, Iser BPM, Sá NNB, Yokota RTC, Moura L, Claro RM, Luz MGC. Tendências temporais no consumo de tabaco nas capitais brasileiras, segundo dados do VIGITEL, 2006 a 2011. Cad. Saúde Pública, Rio de Janeiro, 29(4):812–822, abr, 2013. 23568310

[32] Wünsch-Filho V, Mirra AP, López RVM, Antunes LF. Tabagismo e câncer no Brasil: evidências e perspectivas. Ver Bras Epidemiol 2010; 13(2): 175–87.

[33] Barros SGS, Ghisolfi ES, Luz LP, Barlem GG, Vidal RM, Wolff FH et al Mate (chimarrão) é consumido em alta temperatura por população sob risco para o carcinoma epidermóide de esôfago. Arq. Gastroenterol. [Internet]. 2000 1 [cited 2017 Feb 08].37(1):25–30. Available from: http://www.scielo.br/scielo.php?script=sci_arttext&pid=S0004-28032000000100006&lng=en. http://dx.doi.org/10.1590/S0004-28032000000100006. 10962624 10.1590/s0004-28032000000100006

[34] Szymańska K, Matos E, Hung RJ, Wünsch-Filho V, Eluf-Neto J, Menezes A, Daudt AW, Brennan P, Boffetta P. Drinking of mate and the risk of cancers of the upper area aerodigestive tract in Latin America: case control study. Cancer Causes Control 2010; 21:1799–806. doi: 10.1007/s10552-010-9606-6 20623173 10.1007/s10552-010-9606-6

[35] Lubin JH1, De Stefani E, Abnet CC, Acosta G, Boffetta P, Victora C, Graubard BI, Muñoz N, Deneo-Pellegrini H, Franceschi S, Castellsagué X, Ronco AL, Dawsey SM. Mate drinking and esophageal squamous cell carcinoma in South America: pooled results from two large multicenter case-control studies. Cancer Epidemiol Biomarkers Prev 2014;23: 107–16. doi: 10.1158/1055-9965.EPI-13-0796 24130226 10.1158/1055-9965.EPI-13-0796 PMC3947123

[36] Silva DRM, Curado MP, de Oliveira JC.High incidence of esophageal cancer in central-western Brazil: a migrant effect? Eur J Cancer Prev;22(3): 235–43, 2013 5 doi: 10.1097/CEJ.0b013e3283592c9d 22990005 10.1097/CEJ.0b013e3283592c9d

[37] Islami F, Kamangar F. Helicobacter pylori and esophageal cancer risk: a meta-analysis. Cancer Prev Res 2008;1(5):329–339. 10.1158/1940-6207.CAPR-08-0109 PMC3501739 19138977

[38] Zhuo X, Zhang Y, Wang Y, Zhuo W, Zhu Y, Zhang X. Helicobacter pylori infection and oesophageal cancer risk: association studies via evidence-based meta-analysis. Clin Oncology 2008; 20:757–762. 10.1016/j.clon.2008.07.005 18793831

[39] Xie FJ, Zhang YP, Zheng QQ, Jind HC, Wang FL, Chen M, Shao L, Zou DH et al Helicobacter pylori infection and esophageal cancer risk: an uptaded meta-analysis. World J Gastrenterol 2013;19(36):6098–6107. 10.3748/wjg.v19.i36.6098 PMC3785633 24106412

[40] Jee YH, Shin A, Lee JK, Mo C. Decreases in Smoking-Related Cancer Mortality Rates Are Associated with Birth Cohort Effects in Korean Men. Int. J. Environ. Res. Public Health 2016, 13, 1208; doi: 10.3390/ijerph13121208 27929405 10.3390/ijerph13121208 PMC5201349

[41] Ito Y, Iokan A, Nakayama T, Tsukuman H, Nakamura T. Comparison of Trends in Cancer Incidence and Mortality in Osaka, Japan, Using an Age-Period-Cohort Model. Asian Pacific J Cancer Prev, 12, 879–888. 21790220

[42] Liu SZ, Zhang F, Quam PL, Lu JB, Liu ZC, Sum XB. Time Trends of Esophageal Cancer Mortality in Linzhou City During the Period 1988–2010 and a Bayesian Approach Projection for 2020.Asian Pacific Journal of Cancer Prevention, Vol 13, 2012. 10.7314/apjcp.2012.13.9.4501 23167368

[43] Adair T, Hoy D, Dettrick Z, Lopez AD. Trends in oral, pharyngeal and oesophageal cancer mortality in Australia: the comparative importance of tobacco, alcohol and other risk factors. Journal of Public Health 2011 vol. 35 no. 3. 10.1111/j.1753-6405.2011.00700.x 21627720

[44] Seoane-Mato D, Aragonés N, Ferreras E, Garcia-Pérez J, Cervantes-Amant M, Fernandez Navarro PF, Barriuso RP, López-Abente G. Trends in oral cavity, pharyngeal, oesophageal and gastric cancer mortality rates in Spain, 1952–2006: an age-period-cohort analysis.BMC Cancer. 2014 4 11;14:254 doi: 10.1186/1471-2407-14-254 24725381 10.1186/1471-2407-14-254 PMC4022416

[45] Thuler FP, Forones NM, Ferrari AP. Neoplasia avançada de esôfago–diagnóstico ainda muito tardio. Arq Gastroenterol v. 43 –no.3 –Jul-Set 2006. 10.1590/s0004-28032006000300010 17160236

[46] Liu B, Bo Y, Wang K, Liu Y, Tang X, Zhao Y, Zhao E, Yuan L.Concurrent neoadjuvant chemoradiotherapy could improve survival outcomes for patients with esophageal cancer: a meta-analysis based on random clinical trials. doi: 10.18632/oncotarget.14669 28099899 10.18632/oncotarget.14669 PMC5386772

[47] Mariette C, Piessen G, Triboulet JP. Therapeutic strategies in oesophageal carcinoma: role of surgery and other modalities. The Lancet Oncology. 2007; 8(6):545–553. doi: 10.1016/S1470-2045(07)70172-9 17540306 10.1016/S1470-2045(07)70172-9

[48] Shapiro J, Van Lanschot JJ, Hulshof MC, van Hagen P, van Berge Henegouwen MI, Wijnhoven BP, van Laarhoven HW, Nieuwenhuijzen GA, Hospers GA, Bonenkamp JJ, Cuesta MA, Blaisse RJ, Busch OR, et al Neoadjuvant chemoradiotherapy plus surgery *versus* surgery alone for oesophageal or junctional cancer (CROSS): long-term results of a randomised controlled trial. The Lancet Oncology. 2015; 16(9):1090–1098. doi: 10.1016/S1470-2045(15)00040-6 26254683 10.1016/S1470-2045(15)00040-6

[49] Kranzfelder M, Schuster T, Geinitz H, Friess H and Buchler P. Meta-analysis of neoadjuvant treatment modalities and definitive non-surgical therapy for oesophageal squamous cell cancer. The British journalofsurgery. 2011; 98(6):768–783. 10.1002/bjs.7455 21462364

[50] Gomes R, Nascimento EF; Araújo FC. Why do men use health services less than women? Explanations by men with low versus higher education. Cad. Saúde Pública, Rio de Janeiro, 23(3):565–574, 3, 2007. 10.1590/s0102-311x2007000300015 17334571

[51] Brazil. Ministry of Health. Ordinance. No. 874 of May 16, 2013. Available from:http://bvsms.saude.gov.br/bvs/saudelegis/gm/2013/prt0874_16_05_2013.html.

[52] Souza MC, Vasconcelos AG, Cruz OG. Trends in lung cancer mortality in Brazil from the 1980s into the early 21st century: age-period-cohort analysis.CadSaude Publica. 2012 1;28(1):21–30. 10.1590/s0102-311x2012000100003 22267062

