# Supplementary figures and images for: Inequalities in esophageal cancer mortality in Brazil: Temporal trends and projections
Source: PLoS One. 2018 Mar 19;13(3):e0193135. doi: 10.1371/journal.pone.0193135 (PMC5858754; doi:10.1371/journal.pone.0193135)

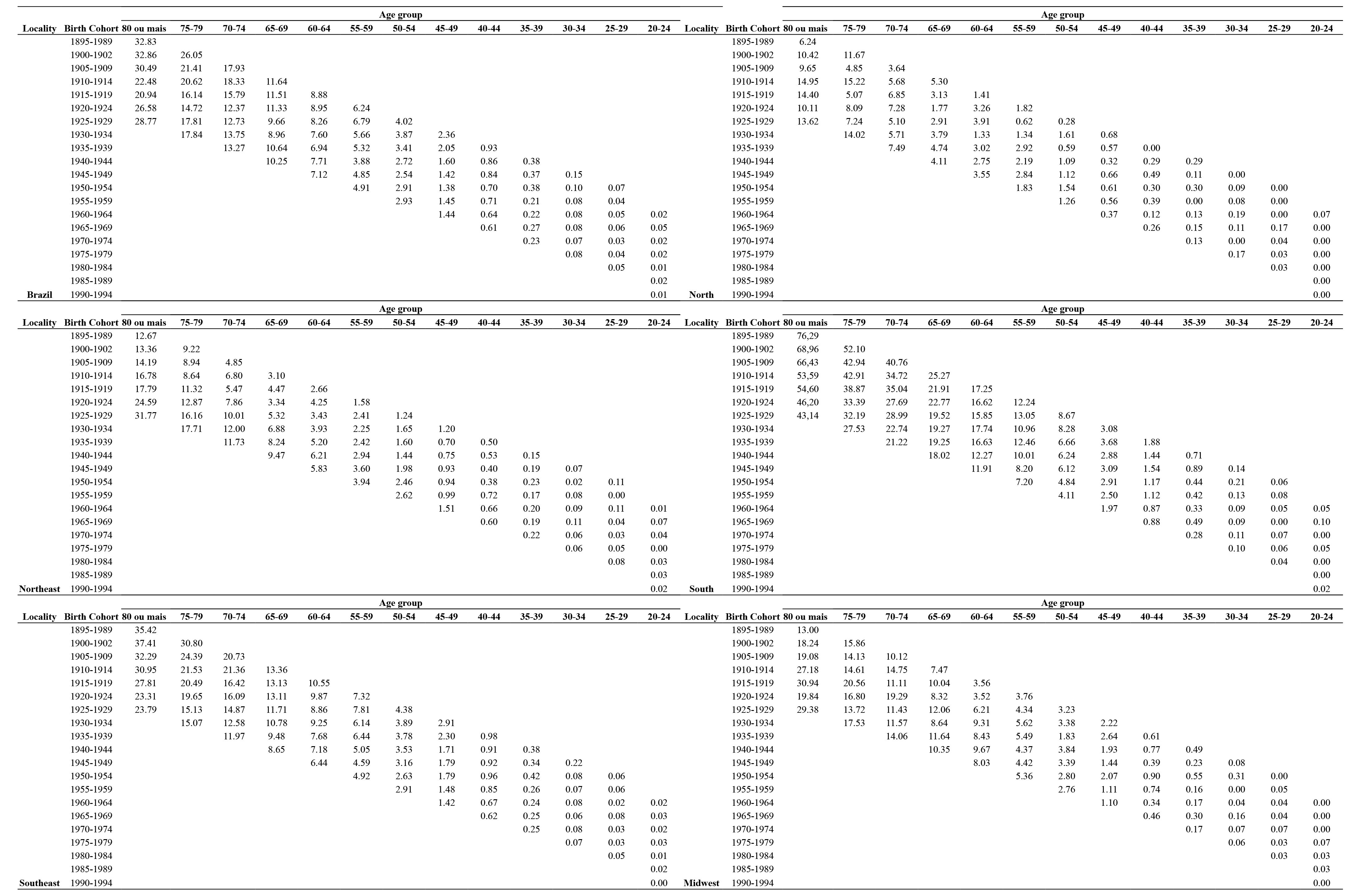

Supplement: S1 Appendix — (TIF) [file pone.0193135.s001.tif]

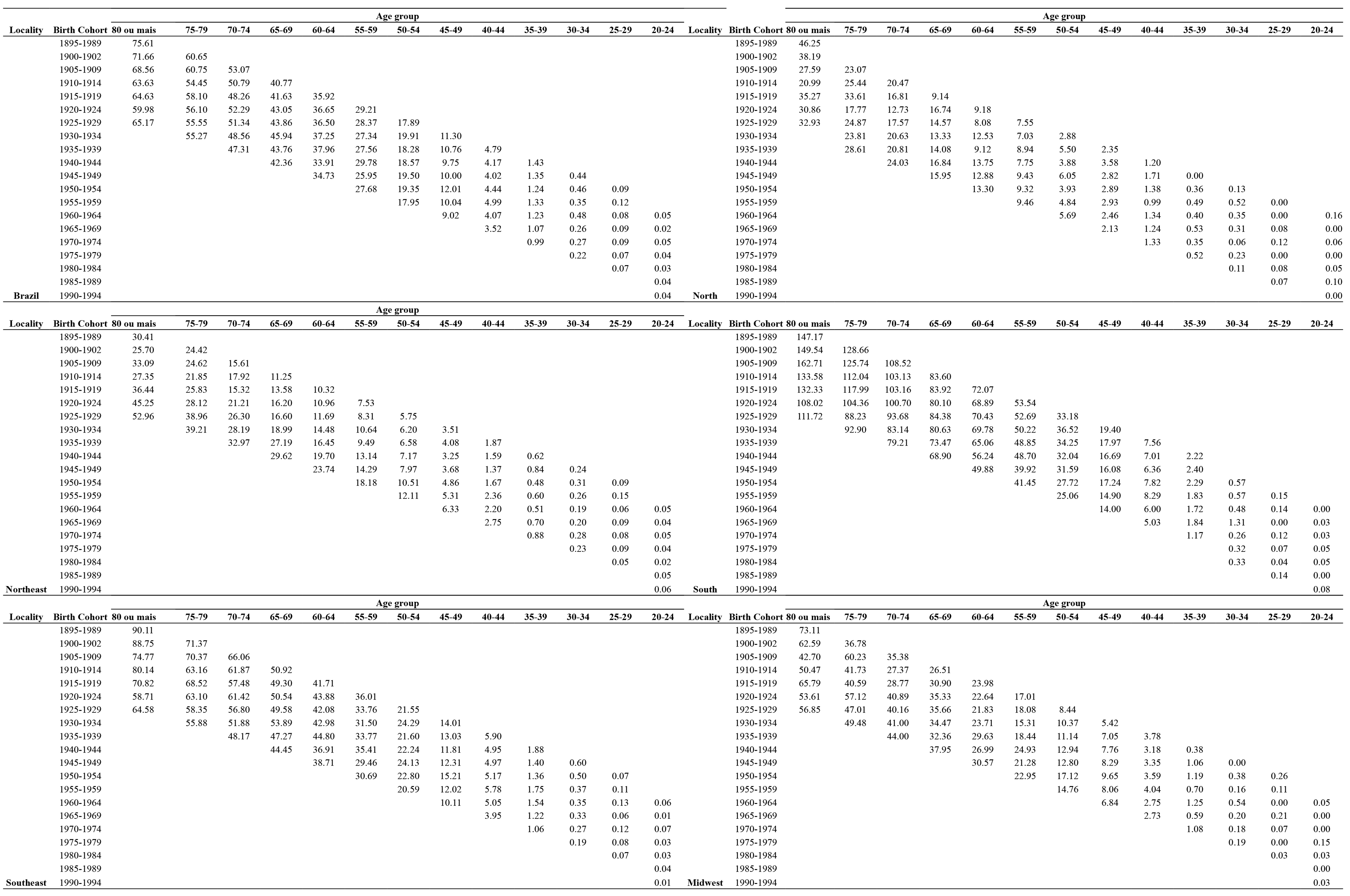

Supplement: S2 Appendix — (TIF) [file pone.0193135.s002.tif]
